# Long-term outcome of dental implants after maxillary augmentation with and without bone grafting

**DOI:** 10.4317/medoral.21055

**Published:** 2016-01-31

**Authors:** Manuel Cara-Fuentes, Jesús Machuca-Ariza, Alberto Ruiz-Martos, Mª-Carmen Ramos-Robles, Ildefonso Martínez-Lara

**Affiliations:** 1 DMD. Private practice, Almeria, Spain; 2 DMD. Private practice, Malaga, Spain; 3 DMD. Private practice, Madrid, Spain; 4 MD, DDS, PhD. Collaborator Professor, Department of Oral Medicine, School of Dentistry, Granada, Spain; 5 MD, PhD. Oral and Maxillofacial Surgeon at University Hospital, Associate Professor of Health Sciences School of Dentistry, Granada, Spain

## Abstract

**Background:**

This study aims to evaluate the technique of sinus bone reformation, which consists of elevating the sinus membrane and placement the implant without bone graft, compared with the widely-used technique involving raising the maxillary sinus and grafting, using animal hydroxyapatite as the filler, while simultaneously fixing the implants.

**Material and Methods:**

This is a retrospective study on two groups of patients who underwent elevation of the sinus membrane and simultaneous placement of the implant. The grafting technique was applied to one group, while the other had no graft. An alveolar ridge height of 4 to 7 mm was necessary. Radiological control was undertaken at 6 months and one year post-prosthetic loading. In each group 38 implants were placed.

**Results:**

No significant behavioural differences were observed in the implants according to the Albrektsson success criteria. Implant failure was observed in 2 implants from the bone grafting group (success rate 93%) and in 1 implant from the reformation group (success rate 97%). In this group, bone formation was observed on both sides of each implant, the bone gain was measured using image management software (2.7±0.9mm mesial and 2.6±0.9mm distal). There was no correlation between mesial and distal bone gain and implant´s length.

**Conclusions:**

The results indicate that bone reformation is a valid technique in cases involving atrophy of the posterior maxilla. Primary stability, maintenance of space by the implant, and the formation of a blood clot are crucial in this technique in order to achieve bone formation around the implant. It is an alternative to the conventional technique of sinus lift with filling material, and has several advantages over this procedure, including a lower infection risk, as it does not involve a biomaterial, reduced cost, a simpler technique, and better acceptance by the patient.

**Key words:**Bone formation, sinus membrane elevation, maxillary sinus, bone grafting.

## Introduction

Implant placement in the posterior maxilla is frequently conditioned by a loss in bone height of the alveolar ridge, the most widespread treatment being the elevation and grafting of the maxillary sinus with different materials.

Currently, the most-used synthetic material is hydroxyapatite, which is slowly resorbed, providing sufficient time for bone maturation and remodelling, maintaining the stability of the space gain in the maxillary sinus through time (1).

A variation on the conventional sinus elevation technique is sinus bone reformation. This consists of sinus membrane raising according to the conventional technique, placement the implant, and placing the membrane over it, without the use of a grafting. It has been found that in the space below the membrane, where the implant is placed, there may be ossification that contributes to bone formation around the implant (2).

In 2003, Lundgren *et al*. (3) described spontaneous bone formation in the maxillary sinus three months after extirpating an intrasinusal cyst, having had to raise the sinus membrane to stitch. In 2006, Palma *et al*. (4) after carrying out experimental studies on goats, showed that the amount of bony tissue increase after elevating the maxillary sinus, either with or without autogenous grafting, is similar after 6 months of healing. In 2007, Thor and Sennerby (5) put implants in the sinus without grafting, suggesting that the titanium surface of the implants demonstrated sufficient thrombogenicity. Activating the coagulation system and platelets affects cell and bone growth.

The implant provides a vertical limit for the upper position of the elevated maxillary sinus membrane, while the space is maintained by the formation of a blood clot (6). Sufficient bone is needed to obtain adequate primary stability. It is thought that when the minimum height is less than 5 mm, there should be a sufficient layer of cortical bone in the sinus floor to achieve stability (7).

Several authors (2,8-10) have obtained satisfactory results with this technique, at least as good as those employing the conventional procedure.

## Material and Methods

This work involves a retrospective study on two groups of clinical cases, where the patients underwent sinus membrane elevation and implant insertion in a single surgical phase. The sinus bone reformation technique, without bone grafting, was applied to one group and 38 implants were inserted. The other group also involved 38 implants, as well as the use of animal hydroxyapatite filler (Bio-oss®, Geistlich Pharma AG, Wolhusen, Switzerland).

The criterion for selecting the surgical technique in the patients was geographical, based on the working location of each of the professionals.

The inclusion criteria for this study were: sinus elevation with bone reformation or grafting and simultaneous insertion of the implants, with an alveolar ridge height of between 4 and 7 mm; availability of at least the initial panoramic radiograph and the 1-year post-prosthetic-loading control radiograph; clinical control of the patient at least a year after the prosthetic loading; no irreparable sinus membrane perforation; and absence of any maxillary sinus pathology. Those cases lacking any of the inclusion criteria were excluded from the study. The patients corresponding to each group were reviewed by the same professionals that carried out the treatments, according to their normal working protocols.

The integration and survival of the implants through time has been evaluated using the success/failure criteria defined by Albrektsson (11) in 1986, applied to each implant individually.

In the reformation group the implants used were Zimmer® SPB, SPWB and TSV, and Phibo®. In the conventional elevation group, Phibo® and Biomet® implants were employed.

This study was performed in accordance with the Declaration of Helsinki. The study was approved by the research ethics committee of the Stomatology Department of the Dentistry School of Granada University, and written informed consent was obtained before participation.

- Surgical technique

All the operations were made under local anaesthetic. A full-thickness crestal incision was made with distal unloading, as well as mesial incision in some cases to avoid the inclusion and injury of the respective papillae. Mucoperiosteal flaps were raised with the precaution of leaving the whole lateral wall of the upper jaw uncovered.

The access window was made with a No. 8 round bur hand piece, always respecting certain limits, locating it at the lower edge, two millimetres above the antral floor. After separating the window with a curette, the integrity of the sinus membrane was examined. In patients with bone reformation, bony windows were always attempted, which could be put back on later. These were made with a piezoelectric motor creating a bevel to allow their subsequent replacement.

In the group using animal hydroxyapatite, Bio-oss®, the implant and filler were put in simultaneously, aiming to infra-prepare in order to achieve greater primary stability of the implants. Antrostomy closure was performed with a resorbable membrane (Bio-Gide®, Geistlish Biomaterials, Switzerland).

**Figure 1:**
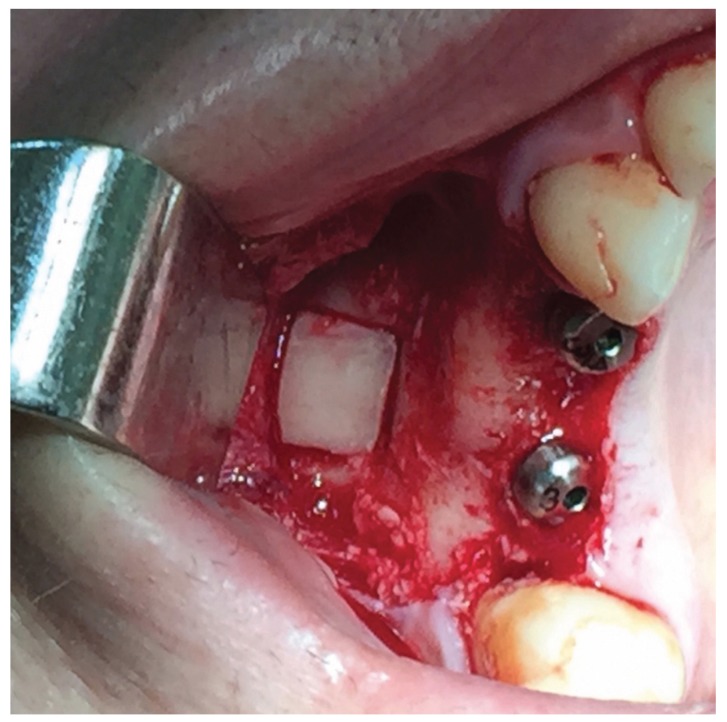
Bony window repositioning after implants placement using bone reformation technique.

In the bone reformation group, after the bony window was removed, the sinus membrane was detached conventionally, including dissection of the palatal wall, to avoid it becoming tense after insertion of the implant. Later, the integrity of the sinus membrane was examined to verify that there was no tearing and the implants were placed, also with infra-preparation. Once fixed, the sinus membrane was placed over the implant and the formation of clotting within the sinus was checked. In most cases it was possible to stably replace the bony window (Fig. 1); a resorbable membrane (Bio-Gide®, Geistlish Biomaterials, Switzerland) was used when replacement was impossible.

- Radiological analysis

When the implants were placed a periapical and/or panoramic x-ray was taken, which was used for measuring the existing residual alveolar height in each case, utilising a specific radiological image management programme (BDSWIN, Dürr Dental, AG, Bietigheim-Bissingen, Germany). It is calibrated to the length of the implant, which is known. It is measured in millimetres from the implant-prosthesis connection (for the Zimmer SPB and SPWB implants we have to take into account the two millimetres corresponding to the polished transepithelial area) up to the last point of bone-implant contact when entering the maxillary sinus, mesially and distally from each implant.

**Figure 2:**
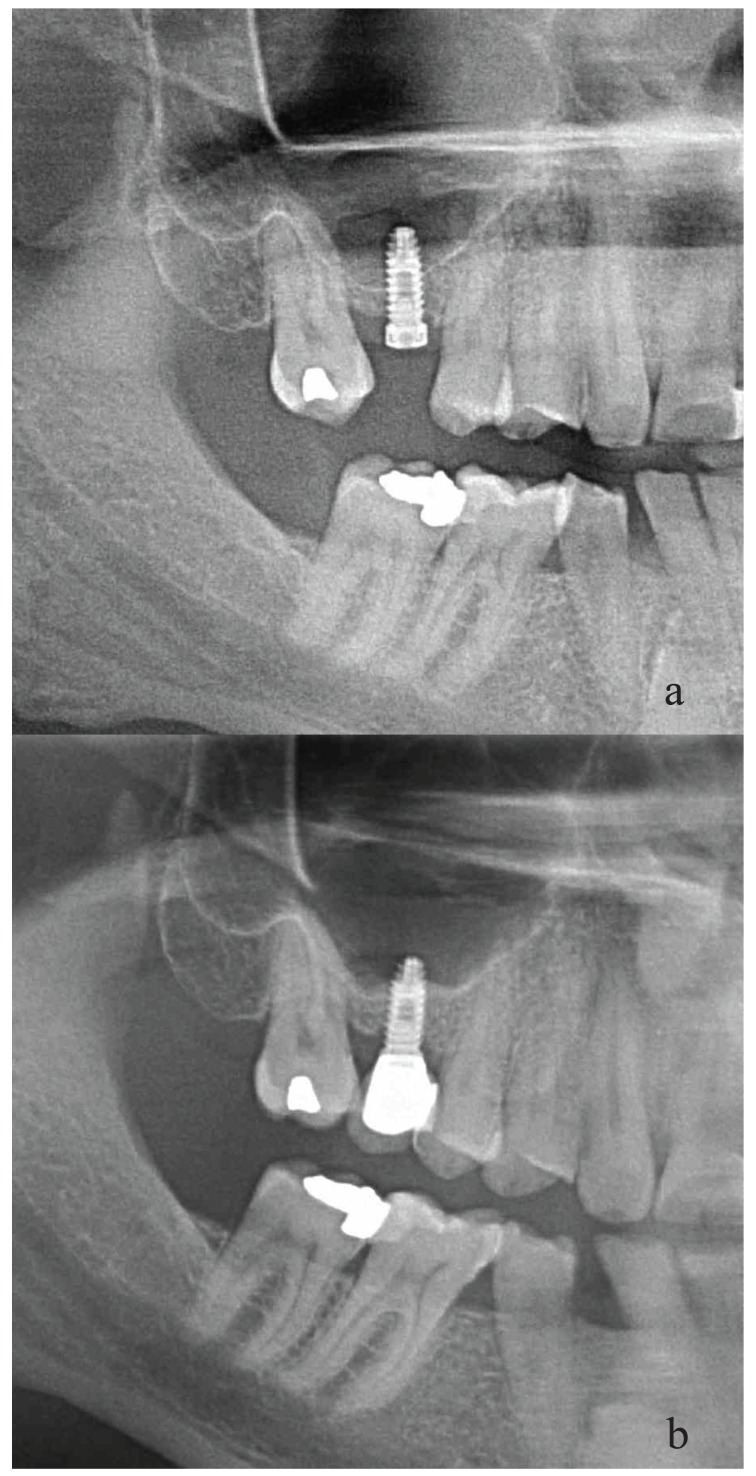
Panoramic view of one implant using sinus bone reformation and bony window repositioning at different times. a. At the time of surgery. b. 36 months after surgery. Bone gain is observed on both sides of the implant.

Radiological control took place 6 months and one year after prosthetic loading. The radiographs enable an assessment of new bone formation around the implants, the degree of resorption of the filler, and the loss of bone at the coronal level in the implants (horizontal and vertical resorption). Bone formation in the reformation cases was also measured with radiological image treatment software (BDSWIN, Dürr Dental, AG, Bietigheim-Bissingen, Germany). The newly formed bone was measured in millimetres. It is measured from the implant-prosthesis join, just as when measuring the residual bone, to the last bone-implant join in the maxillary sinus; the difference from the original measurement is the newly formed mesial and distal bone (Fig. 2 a,b ).

Data graphics and statistical analysis were performed using Prism 5 (GraphPad).  Mann-Whitney tests was applied to evaluate differences between the groups and Spearman correlation coefficient was calculated between bone gain and implant´s length. We used Fisher´s exact test to compared adverse events in the two study groups. For categorical data, the X² test was used. Kaplan-Meier estimates was used to describe the proportion of implants who failed during follow-up. *P* < 0.05 was regarded as statistically significant. Values are presented as means ± s.d. unless otherwise stated.

## Results

The first group comprises 26 patients, 11 men and 15 women aged between 32 and 71, on whom a total of 28 maxillary sinus elevations were performed, with the simultaneous insertion of 38 osseointegrated implants, using the sinus bone reformation technique.

The second group includes a total of 25 patients, 15 men and 10 women aged from 31 to 69, on whom 29 maxillary sinus elevations were performed, with the simultaneous insertion of 38 osseointegrated implants, but grafting the sinus with hydroxyapatite of bovine origin (Bio-Oss®) (Table  Table 1.)

**Table 1:**
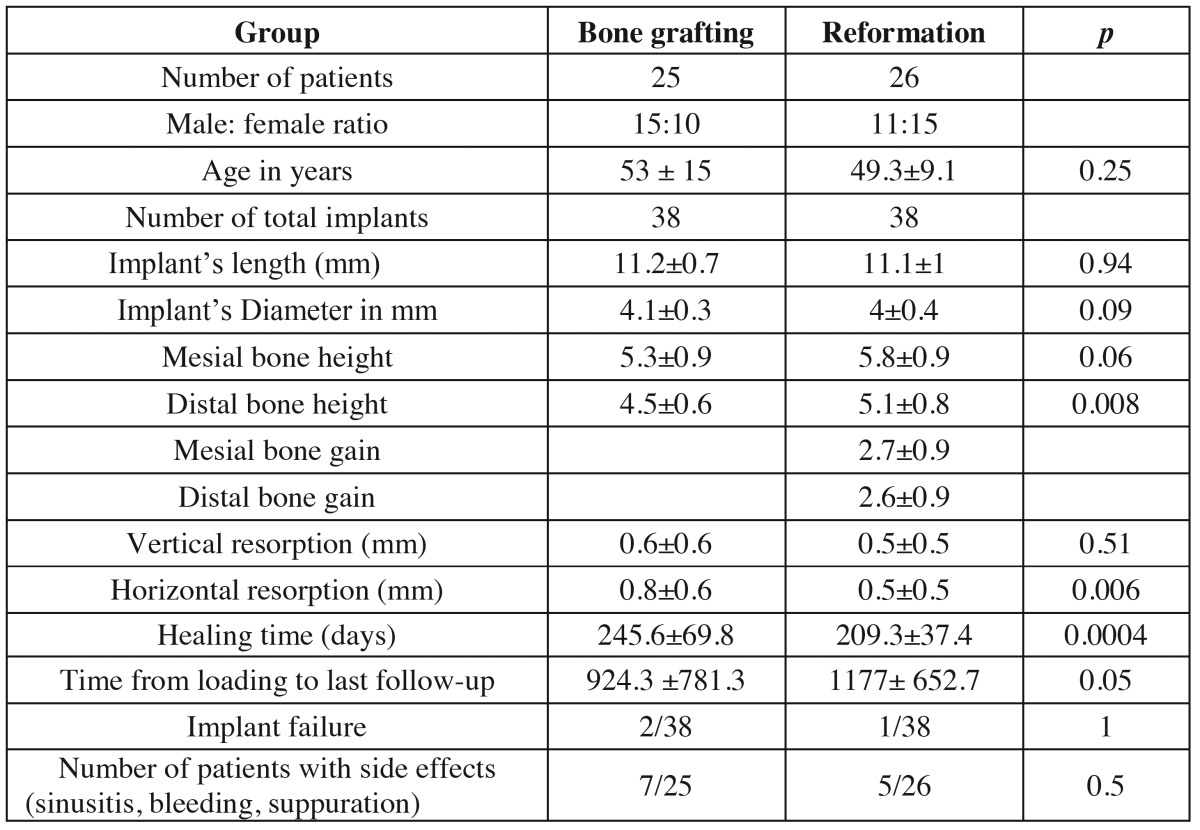
Baseline characteristics of patients and implants.

The mean follow-up time after loading was 924 and 1177 days in the bone grafting and bone reformation group respectively.

**Figure 3:**
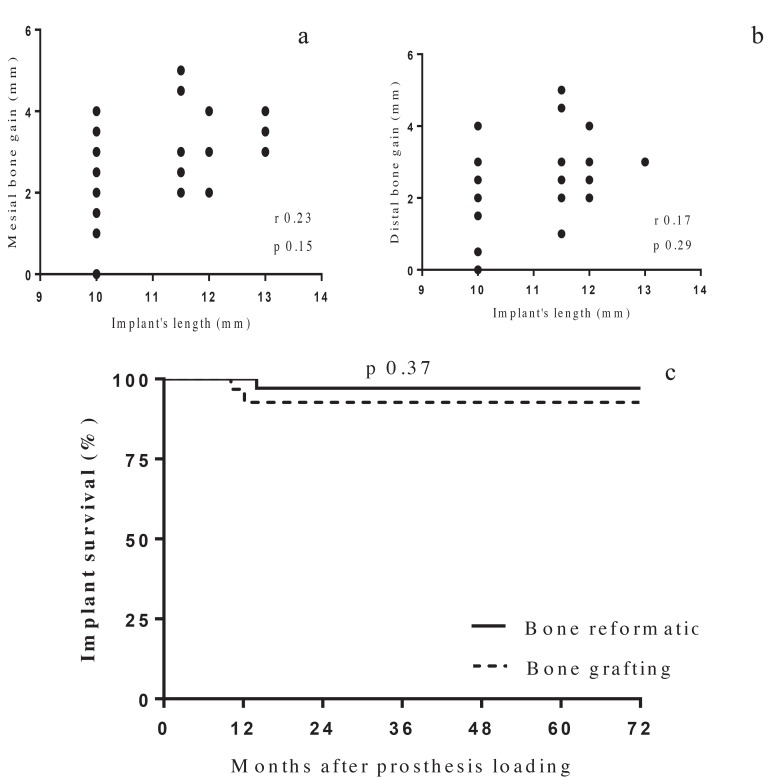
a Bone gain according to implant’s length (mesial) .b. Bone gain according to implant’s length (distal). c. Long-term implant survival. (Kaplan-Meier). Implants at risk Reformation 38 37 30 17 11 6 6 Bone grafting 38 26 16 16 15 7 6

There was no statistical differences between the implant length and diameter among patients who underwent dental implant placement using bone grafting compared to those who underwent bone reformation (*p* 0.94 and 0.09 respectively). The mean mesial and distal bone gain were 2.7 and 2.6 mm respectively in the bone reformation group. Horizontal, but not vertical, bone resorption was significantly higher in patients with bone grafting compared to those in the bone reformation group (*p* 0.0004). In the reformation group, there was no correlation between mesial and distal bone gain and implant’s length (Fig. 3a, b). In addition, bone gain was similar among those patients who had bony window compared to those who underwent closure with resorbable membrane (*p* 0.2).

Implant failure was observed in 2 patients from the bone grafting group and in 1 patient from the bone reformation group. Implant survival at 70 months after loading was similar among patients with bone grafting and those with bone reformation (93% vs 97% respectively) (Fig. 3 c). Side effects (sinusitis, bleeding or suppuration) were observed in 7 of 25 patients in the bone grafting group and in 5 of 26 patients from the bone reformation group. No statistical difference was observed among the two groups.

## Discussion

It is difficult to compare the great number of studies on maxillary sinus elevation techniques available in the literature due to the differences in inclusion criteria, type of implants, patient follow-up, quantity of residual bone present, techniques used, phases of treatment, type of grafting material used, and evaluation methods. Similarly, the definition of success based on histomorphometric parameters is not comparable, as these depend on clinical evolution and short- or long-term response.

In planning the present study, we tried to evaluate the sinus bone reformation technique and, at the same time, compare it with a widely-used technique with predictable results that are increasingly supported in the literature, as is the case of sinus elevation and grafting with hydroxyapatite of bovine origin (Bio-Oss®). In order to decrease the clinical and technical variability, it was decided only to include those cases where the sinus treatment with grafting and implant fixing was carried out in a single phase, as happens in sinus bone reformation. The initial clinical conditions are similar, i.e., the height of the remaining bone theoretically becomes the deciding factor determining the possibility of these procedures, making it feasible to compare the two surgical techniques.

Inserting the implants at the same time as the elevation and sinus grafting is a widely-used technique that is well-documented clinically. Blomquist *et al*. (12) pointed out the advantage of this technique in minimising both costs and surgery time, as well as the fact that the loading can be carried out beforehand, thus maintaining the graft. Many studies (13-15) indicate that there are no clinical or histological differences between the immediate placement of the implant after maxillary sinus elevation and delayed insertion.

The height of the remaining bone is a determining factor when choosing whether to insert the implants in one or two phases, as if there is a height of less than 5 mm there are problems fixing them at the same time as the subantral graft (16,17). In both study groups, most cases involved the positioning of implants with a residual crestal bone height of 5 mm. In certain cases, 4 mm of residual crestal bone was enough to achieve adequate primary stability. No difference was observed between the results obtained according to the available bone height and primary stability is considered more important that the amount of residual crestal bone present. A study has recently been published with even less residual bone in which immediate prosthetic loading was carried out (18).

For various reasons, many of the studies published on the use of the bovine hydroxyapatite Bio-oss® are not useful for comparing with this group. These include delayed implant placement, its use with another filler, no patient follow-up specified, no reference made to the prior bone height in the residual ridge, or no success rate shown. The results obtained in our study are similar to some of those published that were conducted under equivalent conditions (19-22).

Sinus bone reformation offers a series of advantages over the conventional grafting technique: it needs no graft material; a second surgical field is not necessary (if it is autologous bone); there is less morbidity (in the case of autologous bone); a lower infection risk; no risk of the graft material failing; it is cheaper; the technique is simpler; and there is greater patient acceptance when no filler is inserted.

Various theories have been put forth to explain the osteoformation that occurs without the use of a graft. Srouji *et al*. (23) showed that the cells derived from the sinus membrane can grow in culture expressing osteoprogenitor cell markers and osteogenic differentiation can be induced, as well as new bone formation in transplant area. This provides evidence of the presence of osteoprogenitor cells within the Schneider membrane. An important factor in this process is the elevation of the membrane and the exposure of the medial sinus wall, because mesenchymal cells migrate from the exposed sinus wall. Since bone formation requires the recruitment, migration and differentiation of pluripotent mesenchymal cells into osteoblasts, it points to the periosteum of the sinus membrane being another possible source of bone-forming cells (24).

Maintaining the integrity of the maxillary sinus membrane, making sure it stays raised for there to be enough space to build bone, and forming a blood clot, are prerequisites for bone formation in cases of reformation. The space is maintained thanks to the primary stability of the implant onto which the membrane is deposited, creating a limit, and the formation of a blood clot.

Certain studies use different methods to support the sinus membrane and achieve the “tenting” effect, including: the use of the patient’s own venous blood (25), absorbable gelatine sponges (26), equine-collagen sponges (4), fibrin-rich blocks with concentrated growth factors (27), perforated cylindrical device with hydroxyapatite (28), and star- and H-shaped polyactide devices (29).

In most reformation cases the bony window was replaced enabling the maxillary sinus to be maintained as an “isolated” cavity, favouring bone formation according to the principles of guided bone regeneration. As an alternative, resorbable membranes were used. The bone gain was similar in both. Sohn and Cricchio recommend replacing the bony window (26,29), with Sohn pointing out its hypothetical osteoinductive power (26).

For this reason, the reformation fulfils the requirements suggested by Gurtner *et al*. (30) as essential elements for achieving success in bone regeneration: stem cells, anchorage elements, and growth factors.

The results obtained for the reformation group in our work are similar to those of various other studies (2,5,6,8-10). No bone gain differences between mesial and distal side of the implants were found. These results present the technique as a clear alternative to elevation of the maxillary sinus with grafting material; it shows similar results, especially when considering practical issues such as safety and cost. Although the final validation of this technique must undoubtedly come from an analysis of the long-term success of the implants, it is also necessary to understand the intrasinus bone-formation process, particularly those aspects relating to timing, quantity and arrangement, that determine when an implant can receive total functional loading, and the most effective number and arrangement of implants to be fixed in each situation.

After an analysis of the results of the study a series of questions are raised that should be looked at in more extensive future studies. These issues include: where the bone forms preferentially (the implants and/or walls of the maxillary sinus); the timing of the bone formation; the influence of the amount of crestal bone remaining; the importance of the quantity of implants introduced into the maxillary sinus; the effect of replacing the bone access window; and the number of implants necessary to maintain the membrane’s vertical limit.

## Conclusions

1. The results of this study as well as those present in the literature, are similar to, and even sometimes better than, those achieved through the traditional elevation of the maxillary sinus, independent of the bone graft used.

2. The bone reformation can be considered a suitable procedure for inserting implants in cases of posterior maxilla atrophy, where insufficient bone remains for the conventional placement of implants.

3. This technique entails a series of advantages over conventional maxillary sinus elevation with subantral graft: it does not involve grafting, there is less morbidity, a lower infection risk, it is cheaper, and it is better accepted by the patient.

4. Studies involving a larger sample size and longer follow-up period are necessary for determining the factors that influence the degree of bone formation.
